# The Gene *YALI0E20207g* from *Yarrowia lipolytica* Encodes an N-Acetylglucosamine Kinase Implicated in the Regulated Expression of the Genes from the N-Acetylglucosamine Assimilatory Pathway

**DOI:** 10.1371/journal.pone.0122135

**Published:** 2015-03-27

**Authors:** Carmen-Lisset Flores, Carlos Gancedo

**Affiliations:** Department of Metabolism and Cell Signalling, Instituto de Investigaciones Biomédicas “Alberto Sols” CSIC-UAM, Madrid, Spain; University of Leicester, UNITED KINGDOM

## Abstract

The non-conventional yeast *Yarrowia lipolytica *possesses an ORF, *YALI0E20207g*, which encodes a protein with an amino acid sequence similar to hexokinases from different organisms. We have cloned that gene and determined several enzymatic properties of its encoded protein showing that it is an N-acetylglucosamine (NAGA) kinase. This conclusion was supported by the lack of growth in NAGA of a strain carrying a *YALI0E20207g* deletion. We named this gene *YlNAG5*. Expression of *YlNAG5* as well as that of the genes encoding the enzymes of the NAGA catabolic pathway—identified by a BLAST search—was induced by this sugar. Deletion of *YlNAG5* rendered that expression independent of the presence of NAGA in the medium and reintroduction of the gene restored the inducibility, indicating that *Yl*Nag5 participates in the transcriptional regulation of the NAGA assimilatory pathway genes. Expression of *YlNAG5* was increased during sporulation and homozygous *Ylnag5*/*Ylnag5* diploid strains sporulated very poorly as compared with a wild type isogenic control strain pointing to a participation of the protein in the process. Overexpression of *YlNAG5* allowed growth in glucose of an *Ylhxk1glk1* double mutant and produced, in a wild type background, aberrant morphologies in different media. Expression of the gene in a *Saccharomyces cerevisiae hxk1 hxk2 glk1* triple mutant restored ability to grow in glucose.

## Introduction

Hexose kinases initiate the intracellular sugar metabolism in a variety of organisms. In *Saccharomyces cerevisiae* three glucose phosphorylating enzymes encoded by the genes *HXK1*, *HXK2* and *GLK1* have been characterized. The first two encode typical hexokinases while the third one encodes a glucokinase [1, 2]. The lack of growth in glucose of *S*. *cerevisiae* triple mutants *hxk1 hxk2 glk1* indicates that this organism does not possess other enzymes that phosphorylate this sugar [3]. In *S*. *cerevisiae* the gene *HXK2* is expressed at high levels during growth in glucose while *HXK1* and *GLK1* are repressed making Hxk2 the important enzyme for glucose metabolism [4]. Hxk2 also acts as a moonlighting protein regulating the expression of some genes subjected to catabolite repression [5, 6]. Hexokinases and glucokinases have also been shown to participate in signalling pathways in a variety of organisms [7, 8].

Different yeast species exhibit diverse glucose phosphorylating equipments: in *Kluyveromyyces lactis* an hexokinase [9] and a low activity glucokinase are present [10], in *Schizosaccharomyces pombe* there are only two hexokinases [11] while *Hansenula polymorpha* [12] or *Yarrowia lipolytica* [13, 14] have both an hexokinase and a glucokinase. However, in *Y*. *lipolytica* the glucokinase activity accounts for about 80% of the glucose phosphorylating activity during growth in this sugar [14]. *Y*. *lipolytica* is a strictly aerobic, dimorphic yeast that separated early from the common yeast evolutionary trunk and is distantly related to other ascomycetous yeasts [15, 16]. It is receiving increased attention both in basic and applied research due to a series of particular properties. From a basic point of view it has been used to study protein secretion [17], peroxisome biogenesis [18], dimorphism [19] and mitochondrial complexes [20]. Important differences with the model yeast *S*. *cerevisiae* have been shown in some regulatory properties of glycolytic enzymes [21, 22], or in the transcription of certain glucose repressed genes [23, 24]. Also telomeric proteins present in other yeast species are absent in *Y*. *lipolytica* [25]. From a biotechnological point of view this yeast is important in the production of heterologous proteins [26] organic acids [27] or novel biofuels [28, 29].

During a study of the *Y*. *lipolytica* hexose kinases, we found in a comparative BLAST analysis that *Y*. *lipolytica* possesses a putative protein with sequence similarity with a plethora of hexokinases from different origins. The gene encoding it is *YALI0E20207g* and it appeared of interest to elucidate its function as it could reveal the existence of a kinase missed in conventional tests as it occurred for the glucokinase of *K*. *lactis* that allows growth of this yeast in glucose with a doubling time of 30 hours [10]. We have cloned the gene *YALI0E20207g* and biochemically characterized its encoded protein. In this work we present biochemical and genetic evidence showing that the gene encodes an N-acetylglucosamine (NAGA) kinase whose sequence does not show marked similarity with NAGA kinases from other organisms. Expression of the gene under the control of the *YlTEF1* promoter allowed growth in glucose of a *Ylhxk1glk1* double mutant of *Y*. *lipolytica*. We also present results showing that disruption of *YALI0E20207g* abolishes growth in NAGA, hinders sporulation, and causes derepression of the genes encoding the enzymes of the NAGA assimilatory pathway while its overexpression affects morphology in different media.

## Materials and Methods

### Yeast strains and culture conditions

The *Y*. *lipolytica* and *S*. *cerevisiae* strains used in this work are shown in Table 1 and S1 Table respectively. Yeasts were cultured at 30°C in a synthetic medium with 0.17% yeast nitrogen base (Difco, Detroit, MI) 0.5% ammonium sulphate and glucose, fructose, mannose, NAGA, or ethanol at 2% or glycerol at 3% as carbon sources. Liquid cultures were shaken in a girotory shaker at 180 rpm. For plates 2% agar was added. Auxotrophic requirements were added at a final concentration of 20 μg/ml. Transformation was done using the lithium acetate method as described by Barth and Gaillardin [30] for *Y*. *lipolytica* and by Ito *et al*. [31] for *S*. *cerevisiae*. Growth was followed measuring optical density at 660 nm. Mating, sporulation of diploid strains and spore staining by malachite green were carried out as in Flores *et al*. [14].

**Table 1:** *Yarrowia lipolytica* strains used in this work.

Strain	Relevant genotype	Origin
CJM 455 (W29, CLIB 89)	*MatA GLK1 HXK1 NAG5* *	Collection de Levures d'Intérêt Biotechnologique, Thiverval-Grignon, France
PO1a	*MatA ura3 leu2*	C.Gaillardin, INRA.Grignon,France
CJM 936	*MatA ura3 LEU2*	Th. Petit, this laboratory.
CJM 755	*MatB glk1*::*LEU2 hxk1*::*URA3*	C.L. Flores, this laboratory
CJM753	*MatA nag5*::*LEU2 ura 3*	This work
CJM787	*Mat B glk1*::*LEU2 hxk1*::*URA3/* pCL149 (*NAG5*)	This work
CJM 660	PO1a / pCL49L void plasmid, *LEU2* marker	This work
CJM 762	PO1a/ pCL149L (*NAG5*)	This work
CJM 886	*MatA nag5*::*LEU2/* pCL149 (*NAG5*)	This work
CJM 1060	Diploid *GLK1 HXK1 NAG5*	C.L. Flores, this laboratory
CJM 849	Diploid *nag5*::*LEU2*/*nag5*::*LEU2*	This work

* *NAG5* is the name given in this work to gene *YALI0E20207g*

### Nucleic acid manipulations, plasmid constructions and RT-qPCR

Recombinant DNA manipulations were done by standard techniques. Genomic DNA was obtained as described in Hoffman and Winston [32]. Total RNA from the different strains of *Y*. *lipolytica* was obtained using the Speedtools total RNA extraction kit from Biotools B&M Labs S.A.(Spain)

The transcription initiation site of *YALI0E20207g* was determined using a RLM-RACE reaction with the First Choice RLM-RACE kit from Ambion (Life Technologies). To express *YALI0E20207g* in yeasts a 1400 bp piece of DNA comprising 3 bp upstream of the first ATG and 8 bp after the TAG termination codon was isolated by PCR using *Y*. *lipolytica* DNA as template and oligonucleotides 5´-ATATGTCCATGGGAGATGACG and 5´-TATCTACTGTGAAAGCTGGCT. This fragment and all subsequent PCR products were sequenced from both strands. The *YALI0E20207g* DNA fragment was cloned into plasmid pGEM-T Easy (Promega Biotech Iberica, Spain) to give plasmid pCL148, excised with *Not*I and ligated into plasmids pCL49 [23] or pCL49L [14] both carrying the *YlTEF1* promoter, the *YlXPR2* terminator, and *URA3* or *LEU2* respectively as markers for expression in *Y*. *lipolytica*, or in plasmid pDB20 [33] for expression in *S*. *cerevisiae*. The resulting plasmids were named pCL149, pCL149L and pCL150 respectively.

For RT-qPCR the quality of RNA was checked using the Agilent 2100 Bioanalyzer. Only RNAs with a RIN > 9 were used. Non characterized putative genes of the NAGA utilization pathway were identified from the Génolevures database (http://cbi.labri.fr/Genolevures/index.php, [34]) using a BLAST search. The primers used for RT-qPCR are shown in S2 Table. All of them were checked for specificity using the Primer-BLAST from NCBI against the *Y*. *lipolytica* CLIB122 genomic sequence. Total RNA was reverse-transcribed into cDNA using the High Capacity RNA-to-cDNA Kit (Applied Biosystems). The cDNA levels were then determined using a LightCycler 480 from Roche and the LightCycler 480 SYBR Green I Master mix (Roche) with each primer at 250 nM. Each sample was tested in triplicate. After completion of the RT-qPCR melting-curve data were collected to verify PCR specificity, the absence of contamination and primer dimers. Transcripts of the gene YALI*0F27533g* (*ARP4*) were used to normalize the data [14].

### Disruption of *YALI0E20207g*

Plasmid pCL148 was cut with *Stu*I and *Xho*I and blunt ended. This digestion eliminates a fragment of 367 bp internal to the *YALI0E20207g* ORF starting 364 bp after the initial ATG. Plasmid pINA62 [35] was cut with *Nco*I and a fragment of 2.1kb containing the *YlLEU2* gene was excised, blunt ended and ligated with the previous digestion product. The 3.1kb *Not*I fragment of the resulting construct was used to disrupt the chromosomal copy of *YALI0E20207g* in strain PO1a. Disruption was checked by PCR and Southern analysis.

### Purification of YlNAGA-kinase expressed in *Escherichia coli*

Plasmid pCL148 was cut with *Eco*RI and the 1400 bp fragment containing gene *YALI0E20207g* was ligated into plasmid pGEX-4T-2 (GE Healthcare Life Sciences) cut with the same enzyme. *E*. *coli* BL21(DE3) transformed with the resulting plasmid, pCL151, was grown in LB to an OD of 0.5 at 660 nm and then induced with 1mM IPTG for 2 hours and cells were harvested by centrifugation at 12000 x g. Extracts were done by suspending cells in 50 mM phosphate buffer, 1 mM EDTA, 1 mM PMSF, 1mM DTT and 0.1 mM NAGA pH 7.6 and 0.12 mg/ml lysozyme. The suspension was incubated at 37° C for 10 min and centrifuged for 20 min at 27200 x g at 4°C. The supernatant was bound to a Glutathione Sepharose 4B column (GE-Healthcare) and the recombinant protein was eluted with 50 mM glutathione in the buffer described above.

### Enzymatic assays

All assays were carried out at 30°C. Phosphorylation of NAGA, glucose, fructose or mannose by purified protein preparations was assayed spectrophotometrically at 340 nm coupling the production of ADP to the oxidation of NADH in 0.1 M Tris-HCl pH 7.5, 2 mM ATP-Mg, 0.3 mM NADH, 5 mM PEP, and excess pyruvate kinase and lactate dehydrogenase. When Km values were determined the concentration of sugars was varied as needed. When yeast extracts were used, the phosphorylation of glucose, fructose or mannose was assayed by a spectrophotometric assay with glucose-6-P dehydrogenase, phosphoglucose isomerase and phosphomannose isomerase as required [1].Yeast cell free extracts were prepared by breaking the yeast with glass beads in 50 mM phosphate buffer, 1 mM EDTA, 1mM PMSF, 1 mM DTT and 0.1 mM NAGA pH 7.6 in five cycles of 1 min of vortexing and 1 min on ice. The extract was centrifuged at 4°C for 15 min at 20000 x g and the supernatant used for determination of enzyme activities. Apparent Km values were obtained using the Eadie Hofstee or the Lineweaver-Burk plots. Data for all substrates were obtained using at least five different substrate concentrations. Protein was assayed with the commercial BCA protein assay kit (Pierce).

### Measurement of glucose utilization

Cells from a glycerol grown culture were resuspended at 1.5 mg_dry weigth_ / ml in 0.17% YNB without nitrogen source with 25mM glucose and shaken at 30°C. Samples were taken along time; centrifuged one minute at 20000 x g and the supernatant was used for glucose determination using a spectrophotometric assay with NADP, hexokinase and glucose-6-P dehydrogenase.

### Calcofluor staining

Yeasts were collected, washed with water, suspended in calcofluor (1mg/ml) and left in the dark for 10 minutes. Then they were washed twice with water and used for microscopic examination.

### Phylogenetic analysis

Phylogenetic analysis was done using the MEGA6 program [36, 37]. Homologous protein sequences were obtained using NCBI protein blast (http://blast.ncbi.nlm.nih.gov/Blast.cgi). The query coverage was established at > 70% and the E value cut off at 4e^-25.^ Sequences were aligned using MUSCLE [38]. Selection of the best-fit model of amino acid replacement for the phylogenetic analysis was done using ProtTest [39]. The LG+G+I+F model [40] was selected by this software as the best model based on the Akaike information criterion. The maximum likelihood tree was estimated using the MEGA6 software with the LG+G+I+F model. The evolutionary analysis involved 36 amino acid sequences. All positions with less than 95% site coverage were eliminated. There were a total of 406 positions in the final dataset. A bootstrap analysis with 1000 replicates was carried out.

## Results

### Expression of *YALI0E20207g* in a *Y*. *lipolytica* mutant lacking glucose kinases allows growth in glucose and mannose

**Fig 1:**
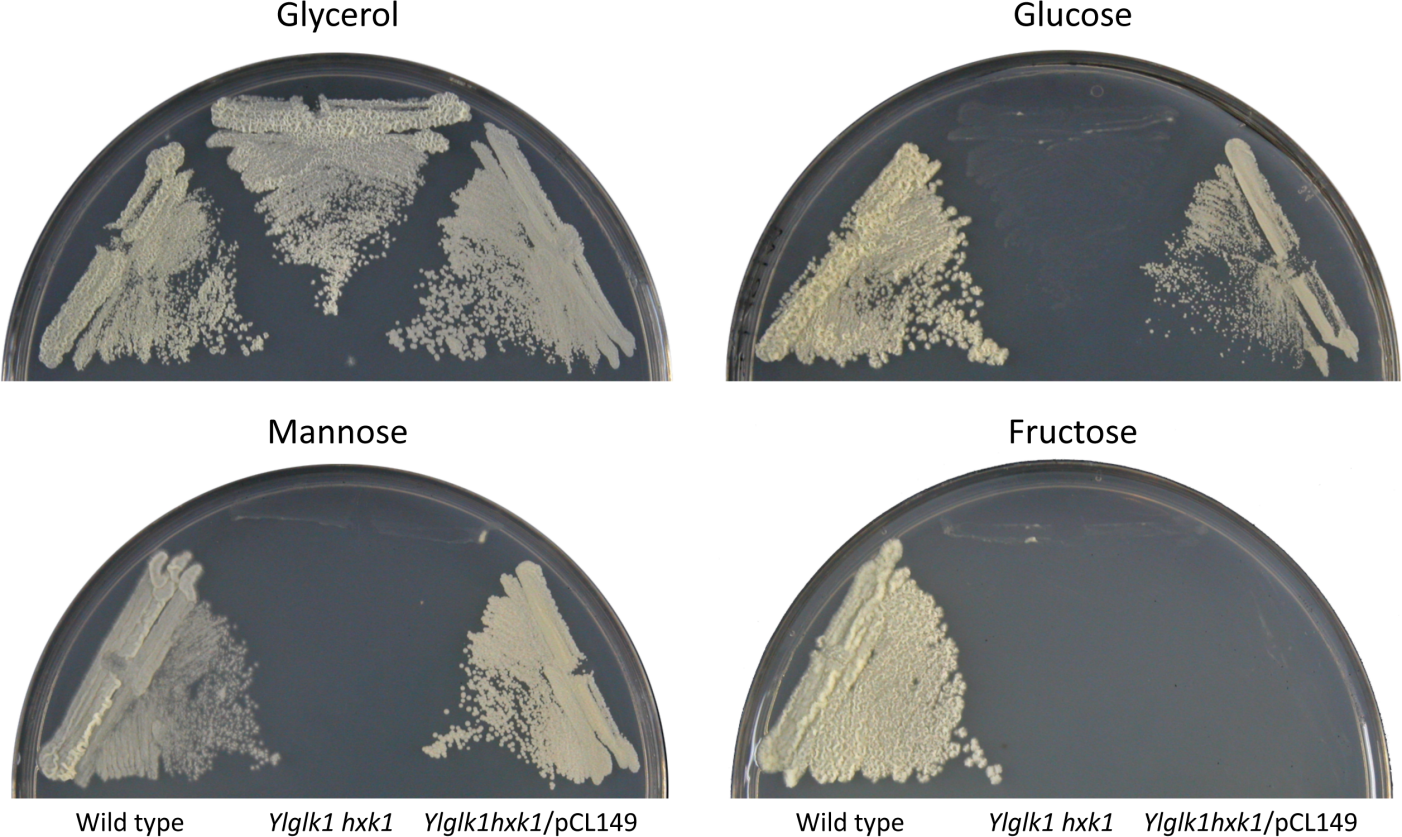
Expression of *YALI0E20207g* complements growth on glucose or mannose of a *Ylglk1 hxk1* double mutant of *Y*. *lipolytica*. *Y*. *lipolytica* strains CJM455, wild type; CJM 755, *Ylglk1 hxk1*; and CJM 787, *Ylglk1 hxk1/*pCL149 (carrying the *YALI0E20207g* gene), were grown on minimal medium glycerol, streaked on minimal medium plates with the indicated carbon sources and incubated at 30°C. Pictures were taken three days after inoculation.

Disruption of the genes encoding glucokinase and hexokinase in *Y*. *lipolytica* results in inability to grow in glucose, fructose or mannose (Fig 1). This result indicates that in this yeast these enzymes are the only ones that allow a significant growth in those sugars. However a BLAST search of the genomic sequence of *Y*. *lipolytica* revealed the existence of a gene, *YALI0E20207g* that could encode a protein with amino acid sequence similarity to yeast hexokinases and glucokinases. To explore the function of this protein, DNA corresponding to its coding sequence was obtained by PCR, cloned, and expressed in an *Ylhxk1 glk1* double mutant of *Y*. *lipolytica*. Since *YALI0E20207g* presents in its 5´region several putative initiation codons we performed before the PCR a RLM-RACE reaction to determine the initiation site of transcription that allowed us to infer the N-terminal sequence of the protein as MSMGDDDRHYHHQMS. When *YALI0E20207g* was expressed under the control of the *YlTEF1* promoter in an *Ylhxk1 glk1* mutant, growth on glucose or mannose but not on fructose was observed (Fig 1). A similar restoration of growth capacity was seen when *YALI0E20207g* was expressed in a *hxk1 hxk2 glk1* triple mutant of *S*. *cerevisiae* unable to grow in those sugars [3] (S1 Fig). Since yeasts glucokinases exhibit activity on glucose and mannose but not on fructose [13, 41], the growth phenotypes indicate that *YALI0E20207g* encodes an enzyme with a sugar specificity similar to that of a glucokinase, that is non operative during growth in glucose.

### ***YALI0E20207g*** encodes a N-acetyl glucosamine kinase

**Fig 2:**
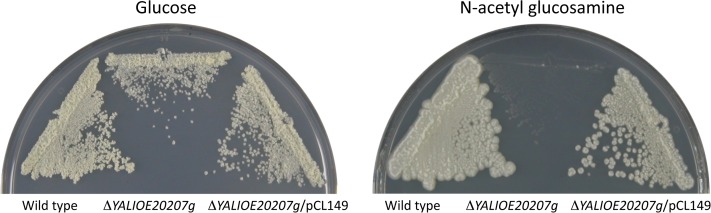
Disruption of *YALI0E20207g* precludes growth on NAGA. *Y*.*lipolytica* strains CJM 445, (wild type); CJM753, with a disruption in *YALI0E20207g* (see Materials and Methods); and CJM 886, (CJM 753 with plasmid pCL149 carrying the wild type gene), grown on minimal medium glycerol, were streaked on minimal medium with NAGA or glucose as carbon sources and incubated at 30°C. Pictures were taken after three days of incubation at 30°C.

The double mutant *Ylglk1 hxk1* overexpressing *YALI0E20207g* grew in glucose slower than a *Y*. *lipolytica* wild type strain and had a glucose utilization rate about 3 times lower than that of a wild type (Table 2). However, determination of its glucose phosphorylating capacity showed no striking difference with that of a wild type (Table 2). These results suggest that the glucokinase activity of YALI0E20207p *in vivo* is low, even when the gene is expressed under the control of a strong promoter, likely due to a low affinity for glucose or ATP. Disruption of *YALI0E20207g* in a wild type strain did not influence its duplication time in glucose or its rate of glucose utilization (Table 2), results consistent with the idea that the main hexose phosphorylating activities responsible for growth in glucose in *Y*. *lipolytica* are *YlGlk1* and *YlHxk1* and that the activity of YALI0E20207p during growth in glucose is low. Determination of the Km for glucose and mannose of the protein YALI0E20207p yielded values of 135 and 67 mM respectively (Table 3), both much higher than those of 0.17 mM for glucose and 1 mM for mannose reported for *Y*. *lipolytica* glucokinase [13] consistent with the previous results. No activity could be detected towards fructose even at high concentrations in the assay, a result in accordance with the observed growth phenotype of the strains expressing only *YALI0E20207g*. The elevated Km values for the sugars assayed suggested that the glucokinase-like activity observed could be due to a marginal activity of a sugar kinase with another physiological function. In some animal tissues, enzymes reported to be glucokinases with a high Km towards glucose turned out to be NAGA kinases with a marginal activity on glucose [42–44]. We found that YALI0E20207p showed high activity towards NAGA with a Km of 0.5 mM (Table 3) suggesting that the protein is a NAGA kinase. Since *Y*. *lipolytica* grows in NAGA it is possible to obtain physiological support for the conclusion reached by the kinetic studies by checking the growth on NAGA of a strain disrupted in *YALI0E202027g*. This disruption abolished growth in NAGA and reintroduction of the gene in the disruptant restored the capacity to grow in this sugar (Fig 2). All these results demonstrate that YALI0E20207p is a NAGA kinase. To avoid multiplication of names for genes encoding proteins with the same biochemical function we will name *YALI0E20207g* as *YlNAG5*, following the designation given to the NAGA kinase encoding gene in *Candida albicans* by Yamada-Okabe *et al*. [45]. An *Ylhxk1glk1* mutant grew in NAGA showing that the corresponding proteins do not participate in the utilization of this sugar.

**Table 2:** Effect of *YALI0E20207g* on duplication time and glucose consumption in *Y*. *lipolytica*.

Strain and relevant genotype	Duplication time (minutes)	Glucose consumption (nmol/min/mg_dw_) ^a)^	Specific phosphorylating activity on glucose (mU/mg protein) ^a)^
CJM936 wild type (*GLK1 HXK1 YALI0E20207g*)	145 ± 14	21.0 ± 1.7	169±3
CJM753 (Δ*YALI0E20207g*)	161 ± 8	21.6 ± 3.7	153±6
CJM755 (*glk1 hxk1*)	no growth	< 1.5	< 2
CJM787 (*glk1 hxk1/p*CL149**-** *YALI0E20207g*)	288 ± 6	7.7 ± 0.1	119 ± 16

^a)^ Strains were grown in minimal medium with glycerol and transferred to minimal medium with glucose. Duplication times and glucose consumption were measured as described in Material and Methods. Total phosphorylating activity on glucose was measured at 600 mM glucose as described in Material and Methods. The results are mean values with standard deviations from three independent cultures.

**Table 3:** Kinetic parameters of the NAGA kinase from *Y*. *lipolytica*.

Substrate	K_m_ (mM)	Relative activity
D-Glucose	135	14
D-Mannose	67	5
D-Fructose	No activity	
NAGA	0.5	100
ATP	1	

The apparent Km values were determined at 30°C by the coupled spectrophotometric method described in Material and Methods using a preparation purified from a GST-*YALI0E20207g* fusion expressed in *E*. *coli*. For the assays with sugars 2mM ATP was used. For the determination of the Km for ATP, 4 mMNAGA was utilized. Similar values for the Km of glucose and mannose were obtained using extracts from the *Y*. *lipolytica* strain CJM787 *glk1 hxk1/*pCL149 (*YALI0E20207g)*. The relative activities were determined at a sugar concentration twice the K_m_ of each sugar. Values are the mean of two determinations with independent preparations.

### The amino acid sequence of the protein encoded by *YlNAG5* differs significantly from that of other NAGA kinases

Once established by function analysis that the protein encoded by *YlNAG5* is a NAGA kinase it appeared interesting to explore why no significant similarity in amino acid sequence with other NAGA kinases was found in the initial BLAST searches. A comparison of the amino acid sequence of *Yl*Nag5 against non-redundant protein sequences databases using a Delta-Blast algorithm produced basically hexokinases or uncharacterized proteins from different origins with about 30% identity and sequence coverages of about 70%. In *Yl*Nag5 the hexokinase pfam03727 domain is present. In this domain we found two amino acid stretches, GTGIN and NCEASLF that were conserved in alignments with other hexokinases. The sequence of the first stretch is comprised in the phosphate 2 region of the ATPase domain of sugar kinases described by Bork *et al*. [46]. Considering that the second stretch is very close to the first one in the primary structure of the protein it is likely that it forms part of this domain too. We introduced changes in these sequences to ascertain their functional importance in *Yl*Nag5. Expression in a *Y*. *lipolytica* strain *nag5*::*LEU2* of plasmids with the mutated variants GT^266^AGIN or NCE^294^QASLF (confirmed by RT-qPCR) did not restore the ability to grow on NAGA to the mutant strain showing the importance of these regions in YlNag5.

**Fig 3:**
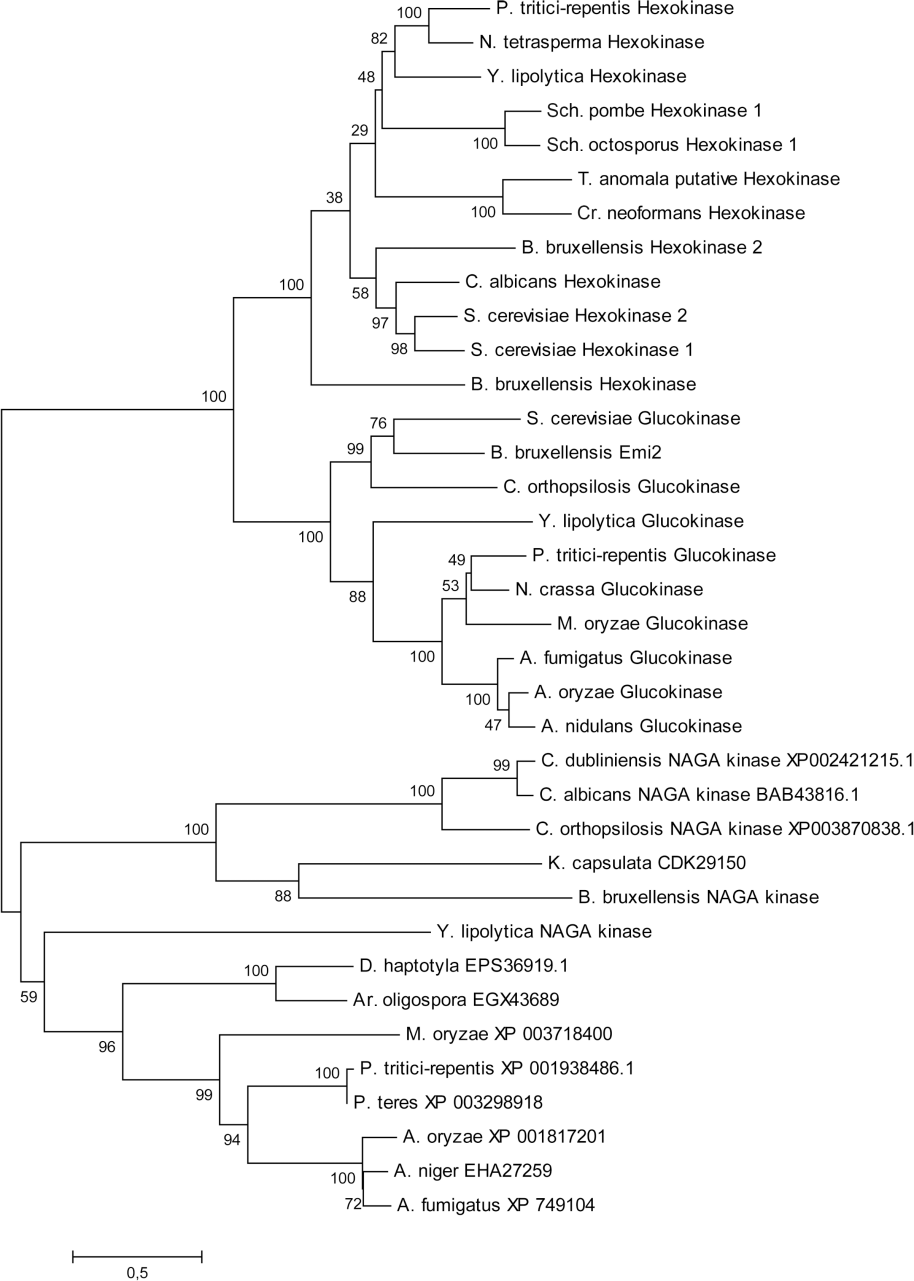
Unrooted phylogenetic tree of NAGA and hexose kinases. A maximum likelihood phylogenetic tree was generated using the MEGA6 software with the LG+G+I+F model as described in Materials and Methods. The bootstrap values are expressed as a percentage shown at key nodes. The sequences were obtained from the GenBank (NCBI) database and the names of the genera in the tree from top to bottom are: P, *Pyrenophora*; N, *Neurospora*; Y, *Yarrowia;* Sch, *Schizosaccharomyces;* T, *Tilletaria*; Cr, *Cryptococcus*; B, *Bretanomyces* (*Dekkera*); C, *Candida*; S, *Saccharomyces*; M, *Magnaporthe*; A, *Aspergillus*; K, *Kuraishia*; D, *Dactylellina*; Ar, *Arthrobotrys*.

We also performed a sequence similarity search using the FASTA algorithm against the UNIPROTKB/Swiss Prot database but no NAGA kinase was found. When the search was done against the UNIPROT database again the overwhelming majority of the proteins found were hexokinases. Only a sequence annotated as a putative NAGA kinase from the yeast *Dekkera bruxellensis* [47], also named *Brettanomyces bruxellensis*, [48] with a 25.6% amino acid identity appeared in the search. We constructed a phylogenetic tree using homologous sequences of proteins from selected organisms from the Pezizomycotina and Saccharomycotina taxa limiting the proteins to those which showed a query coverage >70% and a cut off E value of 4e^-^25 in a BLAST search (Fig 3 and S2 Fig). The tree showed that *Yl*Nag5 appeared close but separated from a group containing other proteins annotated as NAGA kinases (of which only one has been biochemically characterized). Also many proteins annotated as hexokinases or unnamed proteins appeared near *Yl*Nag5. For these proteins we have marked their accession number since they have not been functionally characterized as hexokinases. Since organisms from the Pezizomycotina and Saccharomycotina taxa exhibited proteins similar to hexokinase and NAGA-kinase it is suggested that the appearance of the NAGA-kinases preceded the separation of these taxa.

### Expression of genes encoding the NAGA assimilatory enzymes is induced by NAGA and becomes constitutive in a *Ylnag5* mutant

**Fig 4:**
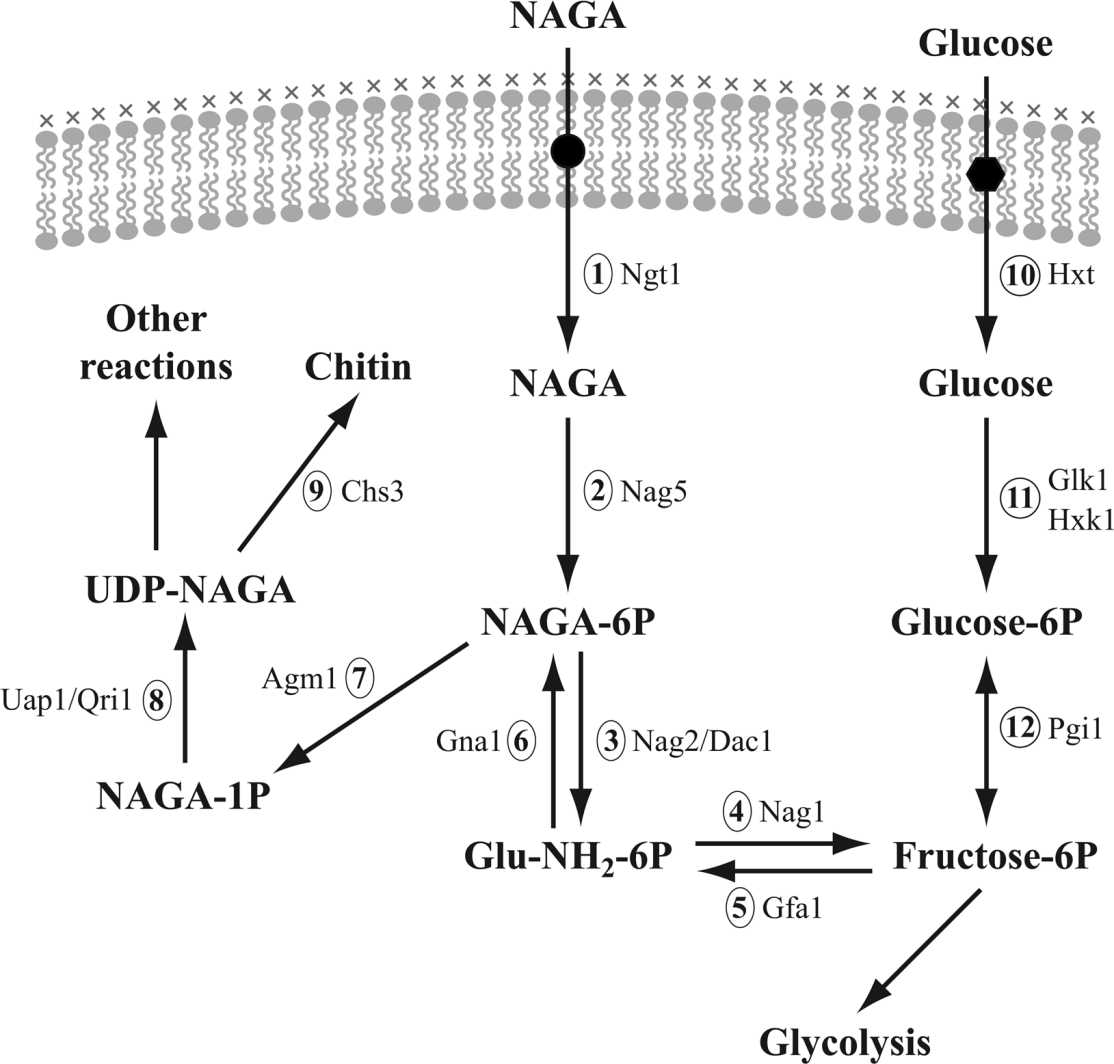
Pathways of NAGA utilization and chitin synthesis in yeasts. The genes of *Y*. *lipolytica* shown were obtained using a BLAST search in the Génolevures database (http://cbi.labri.fr/Genolevures/index.php, [34]) except *YALI0E20207g* that was characterized in this work and hexose kinases as indicated below. The numbers 1–12 refer to the reactions shown in the figure. The names of the genes, those of the putatively encoded or characterized proteins and the assignments in Génolevures are: 1) *NGT1*, NAGA transporter, *YALI0D09801g*; 2) *NAG5*, NAGA kinase, *YALI0E20207g* (functionally characterized in this work; 3) *NAG2*/*DAC1*, NAGA-6P deacetylase, *YALI0E20163g*; 4) *NAG1*, glucosamine-6P deaminase, *YALI0C01419g*; 5) *GFA1*, glutamine-fructose-6P transaminase, *YALI0B21428g*; 6) *GNA1*, glucosamine-6P acetylase, Y*ALI0D20152*g; 7) *AGM1*, NAGA-6P isomerase, *YALI0E29579g*; 8) *UAP1*/*QRI1*, NAGA-1P uridyl transferase, *YALI0E03146g*; 9) *CHS3*, chitin synthase, *YALI0C24354g*; 10) Glucose transporter(s), *YALI0F19184g*, *YALI0C06424g*, *YALI0F06776*, *YALI0B06391*, *YALI0B01342* or *YALI0C08943* [50]; 11) *GLK1*, glucokinase, *YALI0E15488g* [14], or *HXK1*, *YALI0B22308g* [13]; 12) *PGI1*, phosphoglucose isomerase, *YALI0F07711g*.

**Fig 5:**
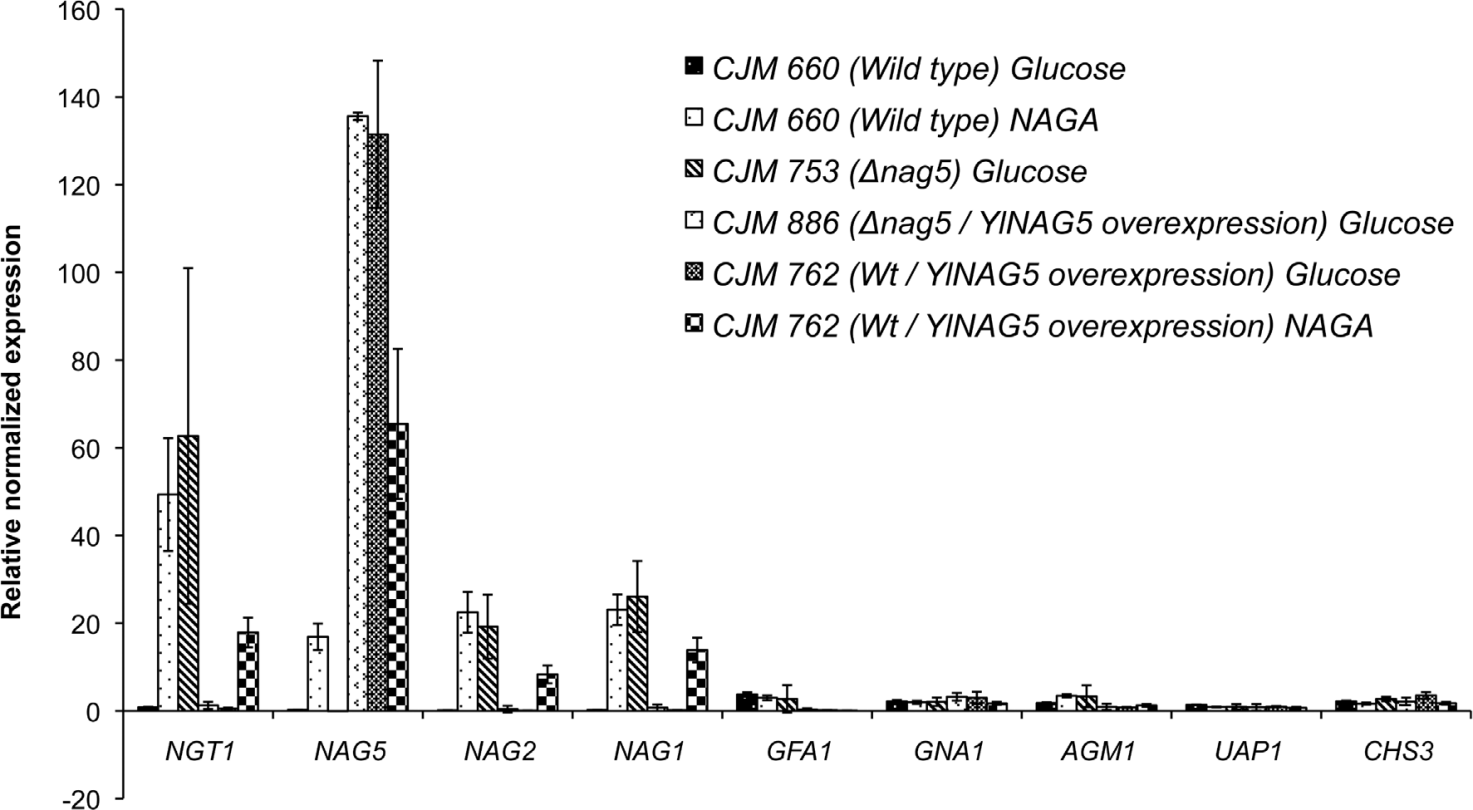
mRNA levels corresponding to genes encoding enzymes of the NAGA pathway and chitin synthesis in *Y*. *lipolytica*. CJM660 (PO1a/pCL49L, void plasmid) CJM762 (PO1a/pCL149L-*YlNAG5*); CJM753 (PO1a*Ylnag5*:*LEU2*); CJM886 (PO1a*Ylnag5*:*LEU2/*pCL149*-YlNAG5*); were grown in minimal medium with glucose or NAGA (except CJM753) as described in Materials and Methods. mRNA levels were quantified by RT-qPCR as described in Materials and Methods. Two independent cultures of each strain were analyzed and three technical replicas were done for each run. mRNA levels were normalized using those of the *YlARP4* gene [14]. The columns represent the mean values of the biological experiments with bars indicating the actual values in the experiments. The gene names correspond to those of Fig 4.

A possible explanation for the lack of growth in glucose of a double *Ylglk1 hxk1* mutant in spite of the presence of the chromosomal copy of *YlNAG5* could be that the expression of this gene is negligible during growth in this sugar. Therefore we examined the levels of expression of this gene and that of the other genes encoding the enzymes of the pathway of NAGA utilization (Fig 4) during growth in glucose and in NAGA. In addition we determined those levels for the genes encoding the enzymes leading from fructose-6-phosphate to chitin since the important intermediate UDP-NAGA is formed also during catabolism of other sugars. The corresponding genes were identified in the genome of *Y*. *lipolytica* by sequence homology using the Génolevures database [34]. As shown in Fig 5 all the genes implicated in the utilization of NAGA were expressed at a very low level during growth in glucose while their expression increased between 20 to 40 times in NAGA grown cultures. A similar behaviour has been reported for the genes *NAG1*, *NAG2*/*DAC2* and *NAG5* in *C*. *albicans* [45, 49]. The genes encoding proteins of the pathway from fructose-6P to chitin (Fig 5) were expressed at similar levels in glucose or NAGA grown cultures suggesting a comparable need for those enzymes in different culture conditions.

We found that a strain with a disrupted *YlNAG5* gene grown in glucose showed an expression of all the genes encoding the enzymes for NAGA utilization similar to those found in the wild type grown in NAGA (Fig 5). Expression of *YlNAG*5 itself followed the same pattern as shown by the values of β-galactosidase expressed from a fusion of the promoter of *YlNAG5* to *E*.*coli lacZ* measured in a wild type and a disrupted strain (Wild type grown in glucose or ethanol <2 mU/mg protein, grown in NAGA 31 mU/mg protein, Δ*YlNAG5* grown in glucose 28, grown in ethanol or acetate 32 mU/mg protein). Reintroduction of *YlNAG5* in a *Ylnag5* background restored the repression by glucose to the genes of the assimilatory pathway (Fig 5) suggesting that the protein *Yl*Nag5 participates in the control of the expression of the genes implicated in the NAGA assimilatory pathway. In *C*. *albicans* it has been reported that expression of the genes *NGT1* and *NAG1* encoding NAGA transport and NAGA deacetylase respectively was higher in a double mutant *hxk1*/*hxk1* than in a wild type grown in glucose or glycerol (*NAG5* is referred to as *HXK1* in that article) [51]. Disruption of *YlNAG5* did not affect the expression of the genes of the pathway from fructose-6P to chitin (Fig 5) indicating that the effect of *Yl*Nag5 is restricted to the NAGA utilization pathway. Overexpression of *YlNAG5* in a wild type background did not influence repression by glucose of the genes of the NAGA assimilatory pathway but it decreased the levels of expression of those genes on NAGA.

### Other effects produced by the disruption or overexpression of *YlNAG5* in *Y*. *lipolytica*

**Fig 6:**
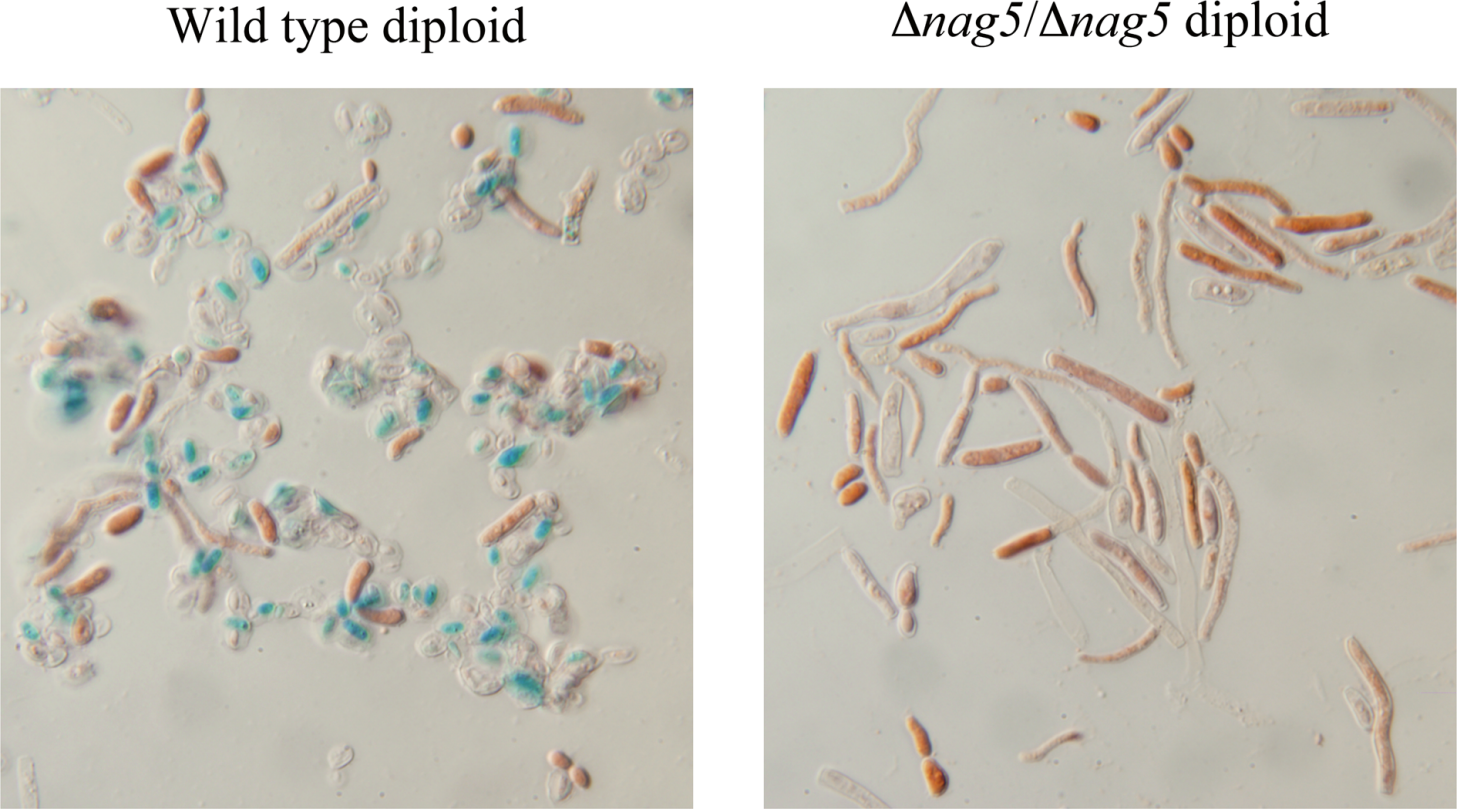
Impairment of sporulation in an homozygous diploid *Ylnag5* mutant. A wild type diploid (CJM 1060) and the isogenic mutant diploid *Ylnag5/Ylnag5* (CJM 849) were streaked in sporulation medium (see Material and Methods) and examined after 8 days. Spores were stained with malachite green and counterstained with safranin (see Material and Methods). Magnification 100x.

A diploid strain carrying disruption of both copies of *YlNAG5* sporulated very poorly as compared with a wild type isogenic diploid (Fig 6). Expression of *YlNAG5* in a wild type diploid, assayed by RT-qPCR, showed an increase after transfer of the strain to sporulation medium (1.5 times after 8 days and 2.5 after 14 days) thus suggesting a role for *Yl*Nag5 in the sporulation process.

**Fig 7:**
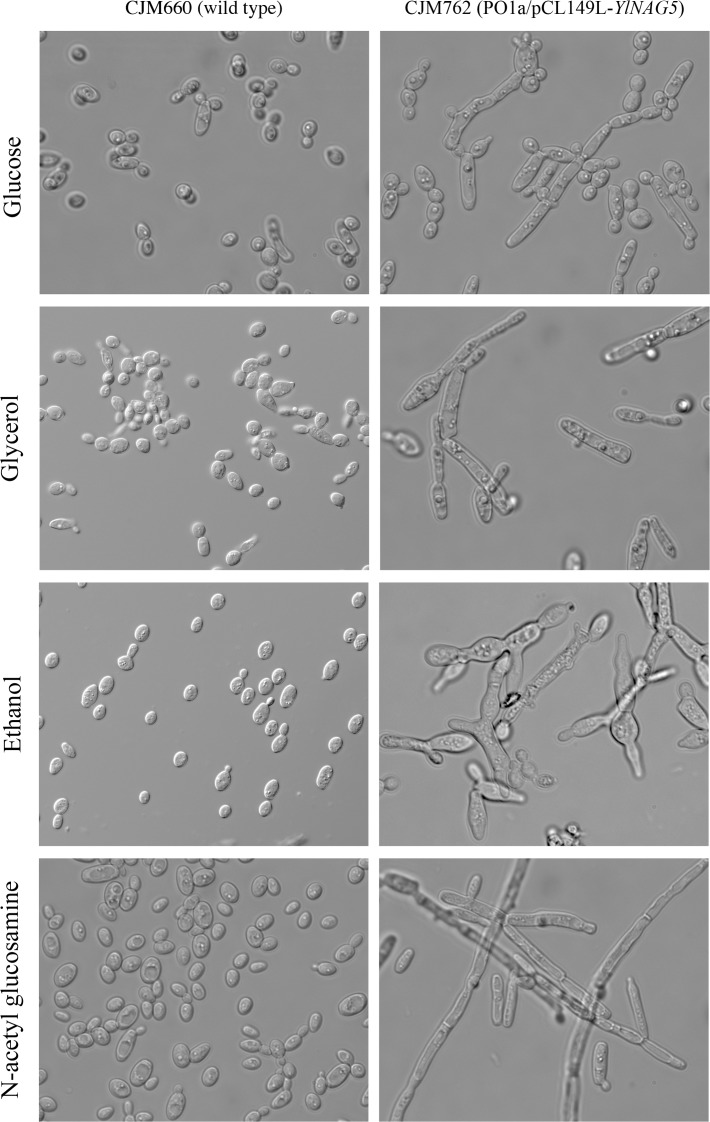
Morphology of a *Y*. *lipolytica* strain overexpressing *YlNAG5* in different media. Strains CJM660 (PO1a/pCL49L, void plasmid) and CJM762 (PO1a/pCL149L-*YlNAG5*) were grown in minimal media with the indicated carbon sources. Pictures were taken with a 100x magnification.

**Fig 8:**
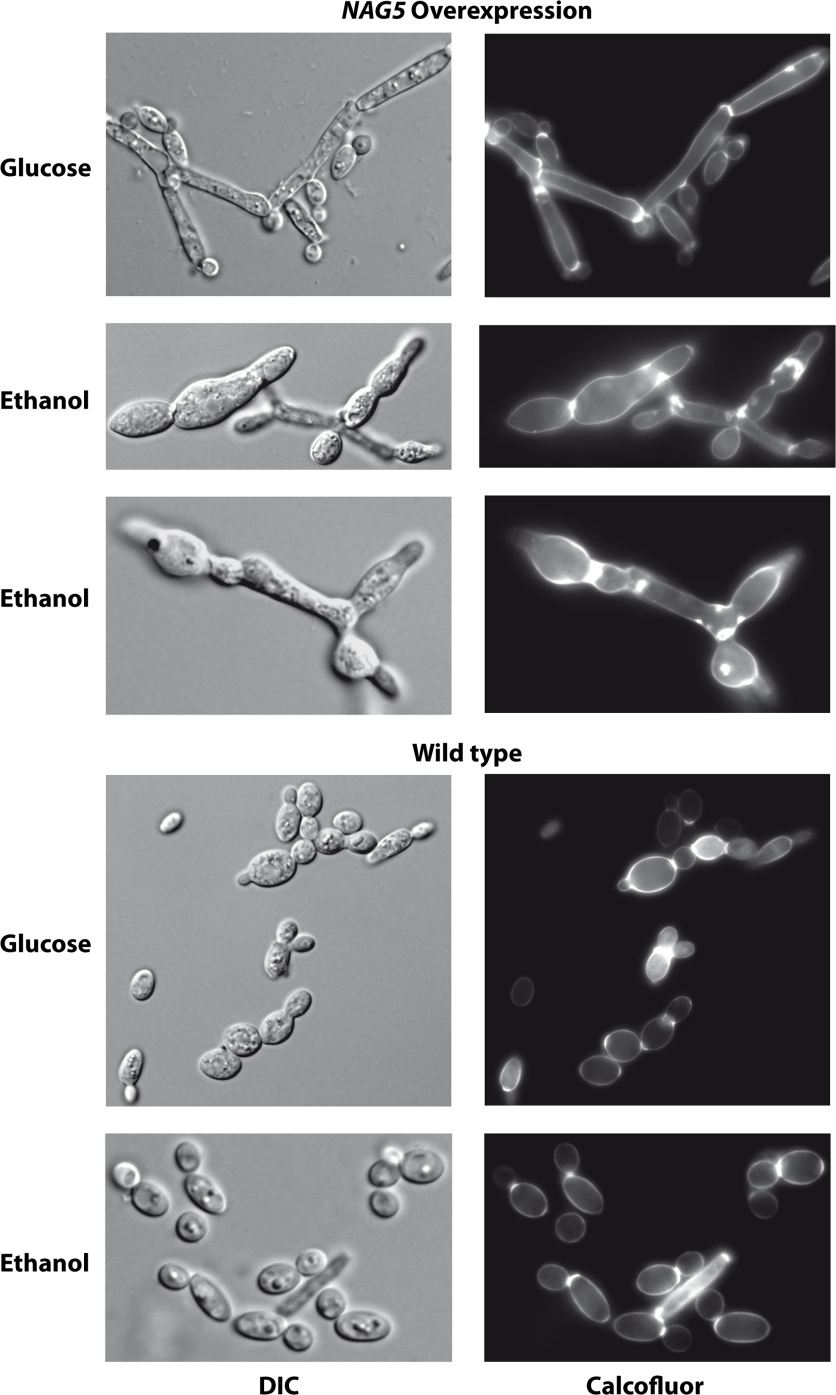
Calcofluor staining of a *Y*. *lipolytica* strain overexpressing *YlNAG5*. Strains CJM660 (PO1a/pCL49L, void plasmid) and CJM762 (PO1a/pCL149L-*YlNAG5*) were grown in minimal medium with the carbon sources indicated and treated with Calcofluor as indicated in Material and Methods. Magnification 100x. DIC (Differential interference contrast).

A strain overexpressing *YlNAG5* exhibited a longer latency to start growth in NAGA when inoculated from a glucose culture as compared with a wild type (400 vs. 200 minutes). This increased lag may be due to an initial overflow of the pathway leading to a transitory depletion of ATP. Another feature of the *Y*. *lipolytica* strain overexpressing *YlNAG5* was its altered morphology. *Y*. *lipolytica* grows in several media as a mixture of yeast-like cells and short hyphae; however the strain overexpressing *YlNAG5* produced cells with elongated or abnormal morphology in different carbon sources (Fig 7). Calcofluor staining showed no striking differences in chitin accumulation between wild type cells and those overexpressing *YlNAG5* (Fig 8). However, changes in the cell wall properties might have occurred in the strain overexpressing *YlNAG5* as shown by the more voluminous, less compact sediment produced by this strain as compared with a wild type grown in similar conditions (S3 Fig)

### Expression of *YlNAG5* in *S*. *cerevisiae* does not produce catabolite repression of the *SUC2* gene

Catabolite repression of the gene *SUC2* encoding invertase in *S*. *cerevisiae* requires the presence of the Hxk2 protein [6]. Since expression of *YlNAG5* in a *S*. *cerevisiae hxk1 hxk2 glk1* mutant restored growth in glucose we tested if it also could substitute for Hxk2 in the glucose repression of *SUC2*. No restoration of catabolite repression of the gene *SUC2* was observed in the strain tested. Also growth on glucose of that strain did not occur in the presence of the respiratory inhibitor Antimycin A indicating a decreased glycolytic flux insufficient to sustain fermentation and making respiration necessary for the use of glucose.

## Discussion

Evidence from enzymatic and genetic tests showed unequivocally that the gene *YALI0E20207g* from *Y*. *lipolytica* encodes the unique N-acetylglucosamine kinase of this yeast. The Km values for glucose and ATP are in the same range as those reported for NAGA kinases from diverse origins [45, 52–54]. The low affinity for glucose of the *Y*. *lipolytica* enzyme is also characteristic of mammalian NAGA kinases that were initially described as glucokinases with low glucose affinity [42, 43]. The only enzymes described with a similar affinity and V_max_ for NAGA and glucose are the RokA hexokinase from *Bacteroides fragilis* [55] and the hexokinase from the archeon *Sulfobolus tokadai* [56]. No activity on fructose has been reported for NAGA kinases and this was also the case for the protein of *Y*. *lipolytica*. The abolition of growth in NAGA in a mutant disrupted in that gene supports the conclusion of the enzymatic tests. We have named the gene *YALI0E20207g NAG5* following the nomenclature of Yamada-Okabe *et al*. [45] for the *C*. *albicans* gene and not *HXK1* as used in the Candida Genome Database to avoid confusion with the name usually employed to designate hexokinases in different organisms and because *HXK1* is already used in *Y*. *lipolytica* [13].

It is interesting to notice that the sequences of NAGA kinases from different organisms biochemically characterized as such often fail to show extensive similarity among them [53, 54, 57, 58]. This is also the case of the NAGA kinase of *Y*. *lipolytica* that showed more sequence similarity with hexo- or glucokinases than with NAGA kinases of other origins. Omelchenko *et al*. [59] have proposed the denomination of non-homologous isofunctional enzymes for enzymes that catalyze the same reaction but that do not show detectable sequence similarity; many NAGA kinases appear to fit in this category. From these considerations and the situation in the phylogenetic tree it could be speculated that several proteins that have not been functionally characterized and appear annotated in databases as related to or similar to glucokinase or hexokinase would turn out to be NAGA kinases. Likely evolution from an ancestral, not very specific, sugar kinase originated the branches leading to hexo-gluco kinases and to NAGA kinases. Among the differences between *Y*. *lipolytica* and other yeasts is the fact that many proteins from this yeast are more similar to proteins from organisms belonging to Pezizomycotina than to those from other Saccharomycotina [60]. Our results with the sequence of its NAGA kinase agree with this observation.

NAGA is a component of several abundant polysaccharides such as chitin, murein or hyaluronic acid from which it can be derived by hydrolytic enzymes of different organisms. However, the use of NAGA as carbon source is not widespread among yeasts [15]. Alvarez and Konopka [61] reported that the ability to use NAGA as carbon source has been lost in several yeast lineages due to loss of different enzymes of the assimilatory pathway. Expression of the corresponding missing heterologous genes renders *S*. *cerevisiae* able to use NAGA [62–64].

NAGA kinase is the first intracellular enzyme of NAGA metabolism in *Y*. *lipoytica* and also in *C*. *albicans* [45] and humans [53]. This contrasts with the situation in *E*. *coli* in which the sugar is phosphorylated by the PTS system during transport and where the NAGA kinase function appears restricted to the utilization of internally produced NAGA from the degradation of murein [54]. A recycling role for NAGA from lysosomal degraded glycoproteins or glycolipids appears also as the main function of NAGA kinase in mammalian cells [53].

One of the intermediates of the metabolic pathway of NAGA is glucosamine-6P. In *S*. *cerevisiae* this compound is formed from the non-metabolizable glucose analog glucosamine, that enters the cell via the glucose transporters and is phosphorylated by hexokinase [65–67] but cannot be further metabolized due to the lack of glucosamine-6-P deaminase. It could be expected that *Y*. *lipolytica* would grow in glucosamine since the glucose transport and the hexokinase are present and the glucosamine-6-P deaminase is functional as shown by the growth in NAGA. However glucosamine does not support growth of *Y*. *lipolytica* [15]. Different explanations may be offered for this behaviour the most plausible ones being a low level of hexokinase [14], a low level of glucosamine-6-P deaminase in the absence of NAGA (this work) or a strong dependence of the deaminase on its allosteric activator NAGA-6P as is the case in *E*. *coli* and other organisms [68, 69].

The genes encoding *NAG5*, *NAG1* and *NAG2*/*DAC1*, in *C*. *albicans* are located contiguously in a cluster in chromosome 6 [45]. Moreover, *NAG1* and *NAG5* share a common bidirectional promoter [45, 49]. Looking for possible orthologous clusters in the genome of the yeast *Dekkera bruxellensis* Curtin *et al*. [47] found that in this yeast genes putatively encoding the enzymes Nag5, Nag1 and Nag2, of the NAGA assimilatory pathway were also located nearby in the same chromosome. We have identified in *Y*. *lipolytica* the genes of the NAGA assimilatory pathway by sequence homology and found that *YlNAG5* and *YALI0E20163g* (*NAG2*) are located nearby in chromosome E, separated by *YALI0E20185g* encoding a putative protein of the glycoside hydrolase 3 family. The other genes encoding proteins of NAGA metabolism were scattered in the *Y*. *lipolytica* genome. Taking into account the position of *Y*. *lipolytica* in the Hemiascomycetous yeasts lineage [16] and that pairs of genes sharing a promoter are not easily dissociated by recombination events [70] it could be speculated that *NAG2* and *NAG5* were originally located nearby in the same chromosome and that further rearrangements during evolution have placed *NAG1* in association with them in other species.

Our results show that the genes of the NAGA assimilatory pathway in *Y*. *lipolytica* are induced by NAGA paralleling the situation in *C*. *albicans* [45, 49]. The low level of *YlNAG5* in the absence of NAGA also explains why a double mutant *Ylglk1 hxk1* does not grow in glucose in spite of possessing a NAGA kinase able to phosphorylate glucose. The finding that the disruption of the *YlNAG5* gene abolished the need of NAGA in the medium to induce the expression of the genes encoding all enzymes of the NAGA utilization pathway and that expression of *YlNAG5* restored their inducibility by NAGA indicates that *Yl*Nag5 participates in the control of the expression of the genes of the pathway although the detailed molecular mechanism(s) of its action remains to be elucidated. There are several known instances of enzymatic proteins that inhibit the transcription of their own encoding gene. In *S*. *cerevisiae* and *K*. *lactis* pyruvate decarboxylase inhibits transcription of the *PDC* promoters when pyruvate decarboxylase reaches a certain level [71, 72]. A case in which a protein acts also as a controller of the expression of the other genes of the pathway is that of the galactokinase from *K*. *lac*tis. The genes encoding the enzymes of the Leloir pathway are induced by galactose and the galactokinase protein is needed to relieve the inhibitory action of the protein Gal80 thus allowing the function of the activator protein Gal4/Lac9 for transcription to proceed [73]. The behaviour of *Yl*Nag5 suggests that it might also behave as a moonlighting protein that participates in the repression of the synthesis of the enzymes of the NAGA utilization pathway. The fact that in *C*. *albicans* the expression of the genes *NGT1* and *NAG1* is increased in the absence of NAGA kinase [51] suggests an important role for Nag5 in the control of the NAGA utilization pathway that has been conserved in distantly related yeast species.

The possible moonlighting role of *Yl*Nag5 in *Y*. *lipolytica* may be a way to regulate the fate of NAGA-6P an intermediate that arises both in the catabolic pathway of NAGA and in that of UDP-NAGA biosynthesis. Simultaneous functioning of the corresponding acetylation/deacetylation reactions and of deamination/amination (Fig 4) could originate futile cycles with detrimental effects to the cell.

The marked negative effect of the disruption of *YlNAG5* on sporulation suggests a role for the protein on the process, an idea supported by the increase in expression of *YlNAG5* when a wild type diploid is placed in sporulation medium. We do not have data yet to hypothesize on the mode of action of *Yl*Nag5.

The increase in the lag phase of growth of the strain overexpressing *YlNAG5* when switched from glucose to NAGA is likely caused by an increased phosphorylation rate that cannot be matched by subsequent reactions to regenerate ATP leading to an initial transitory ATP depletion. In mammals this situation is observed upon a fructose load to the liver; an initial precipitous drop in ATP concentration is followed by a slow phase of recovery that lasts for several hours [74]. Also in *S*. *cerevisiae* the loss of the hexokinase inhibition by trehalose-6-phosphate produces a similar effect [75]. The growth inhibition caused by NAGA in different carbon sources in *E*. *coli* or *C*. *albicans* mutants devoid of NAGA-6P deacetylase or of glucosamine-6P deaminase [76, 77] is likely due to the ATP sink effect of NAGA-6P besides other possible effects of this compound in metabolism.

In addition to its utilization as a nutrient NAGA plays a role in cell signalling in different organisms by various mechanisms (for a review see [78]). NAGA has been used as an external trigger of morphological differentiation in dimorphic yeasts [19, 78, 79]. In the opportunistic pathogenic yeast *C*. *albicans* NAGA induces filamentous growth, a process that appears to have drastic consequences for the invasivity of that organism [80, 81]. The differentiation process is a complex one and elements from different kinase cascades participate in its regulation [82] although with different roles depending on the organism [19]. Rao *et al*. [51] found that homozygous *hxk1*/*hxk1* mutants of *C*. *albicans* (*NAG5* referred to as *HXK1*) presented filamentous growth in media in which a wild type did not form filaments. Alvarez and Konopka [61] reported that a *C*. *albicans* mutant with a deleted *NGT1* gene, that encodes a NAGA transporter, could form hyphae when exposed at very elevated NAGA concentrations suggesting the need for internalization of the sugar to exert its signalling effect(s). Naseem *et al*. [77] using mutants lacking the NAGA catabolic enzymes showed that NAGA induction of morphogenesis is not dependent on its metabolism suggesting that the sugar by itself initiates the signalling pathway(s). The altered morphology of *Y*. *lipolytica* strains overexpressing *YlNAG5* in different media indicates that additional factors different from NAGA play important roles in morphogenesis. In this context it is worth noting that overexpression of NAGA kinase in rat hippocampal neurons upregulated the number of dendrites and increased dendritic branching [83] independently of its enzymatic activity [84] strongly indicating a moonlighting activity of this protein.

## Supporting Information

S1 Fig Phenotypic complementation of growth in glucose of a *S*.*cerevisiae hxk1 hxk2 glk1* strain (CJM 864) by *YALI0E20207g*. Strain CJM 864 was transformed with plasmids pDB20 (void), pCL150 (*YlNAG5*-multicopy), and p381 (*YlNAG5-*centromeric) and streaked in minimal medium with glucose or galactose as carbon sources.
(TIF)

S2 Fig Alignment of the sequences used to build the phylogenetic tree of Fig 3. The alignment generated by MEGA was converted to PIR format using the Format Converter v2.3.5 from the HIV Sequence Database (http://www.hiv.lanl.gov/content/sequence/FORMAT_CONVERSION/form.html) and coloured with the ClustalX color code using Jalview (http://www.jalview.org/).
(TIF)

S3 Fig Fluffiness of the sediment of a *Y*. *lipolytica* strain overexpressing *YlNAG5*. *Y*. *lipolytica* strains CJM660 (PO1a/pCL49L, void plasmid) and CJM762 (PO1a/pCL149L-*YlNAG5*) were grown to an optical density of 10 in minimal medium glucose; 5 ml were transferred to test tubes and photographed after 5h and 24h standing at room temperature. A, CJM660; B, CJM762.
(TIF)

S1 Table *Saccharomyces cerevisiae* strains used in this work. a) Strain CJM 864 was originated as follows: Strains with individual disruptions were crossed, the diploids sporulated and spores were isolated by micromanipulation. Double disruptants of adequate mating type were crossed to select strain CJM 864. This strain was then transformed with the indicated plasmids. b) Plasmid p381 is centromeric and expresses *YALI0E20207g* under the control of the *ScMET25* promoter.
(DOC)

S2 Table Primers used for the RT-qPCR assays. (DOC)
