# Correction: The Gene *YALI0E20207g* from *Yarrowia lipolytica* Encodes an N-Acetylglucosamine Kinase Implicated in the Regulated Expression of the Genes from the N-Acetylglucosamine Assimilatory Pathway

**DOI:** 10.1371/journal.pone.0130113

**Published:** 2015-06-03

**Authors:** The PLOS ONE Staff

## Notice of Republication

This article was republished on May 19, 2015, to correct the legend for Table 3, which was mistakenly incorporated into the body text during the production process. The publisher apologizes for the error. Please download this article again to view the correct version.
