# Supplementary figures and images for: The Gene YALI0E20207g from Yarrowia lipolytica Encodes an N-Acetylglucosamine Kinase Implicated in the Regulated Expression of the Genes from the N-Acetylglucosamine Assimilatory Pathway
Source: PLoS One. 2015 Mar 27;10(3):e0122135. doi: 10.1371/journal.pone.0122135 (PMC4376941; doi:10.1371/journal.pone.0122135)

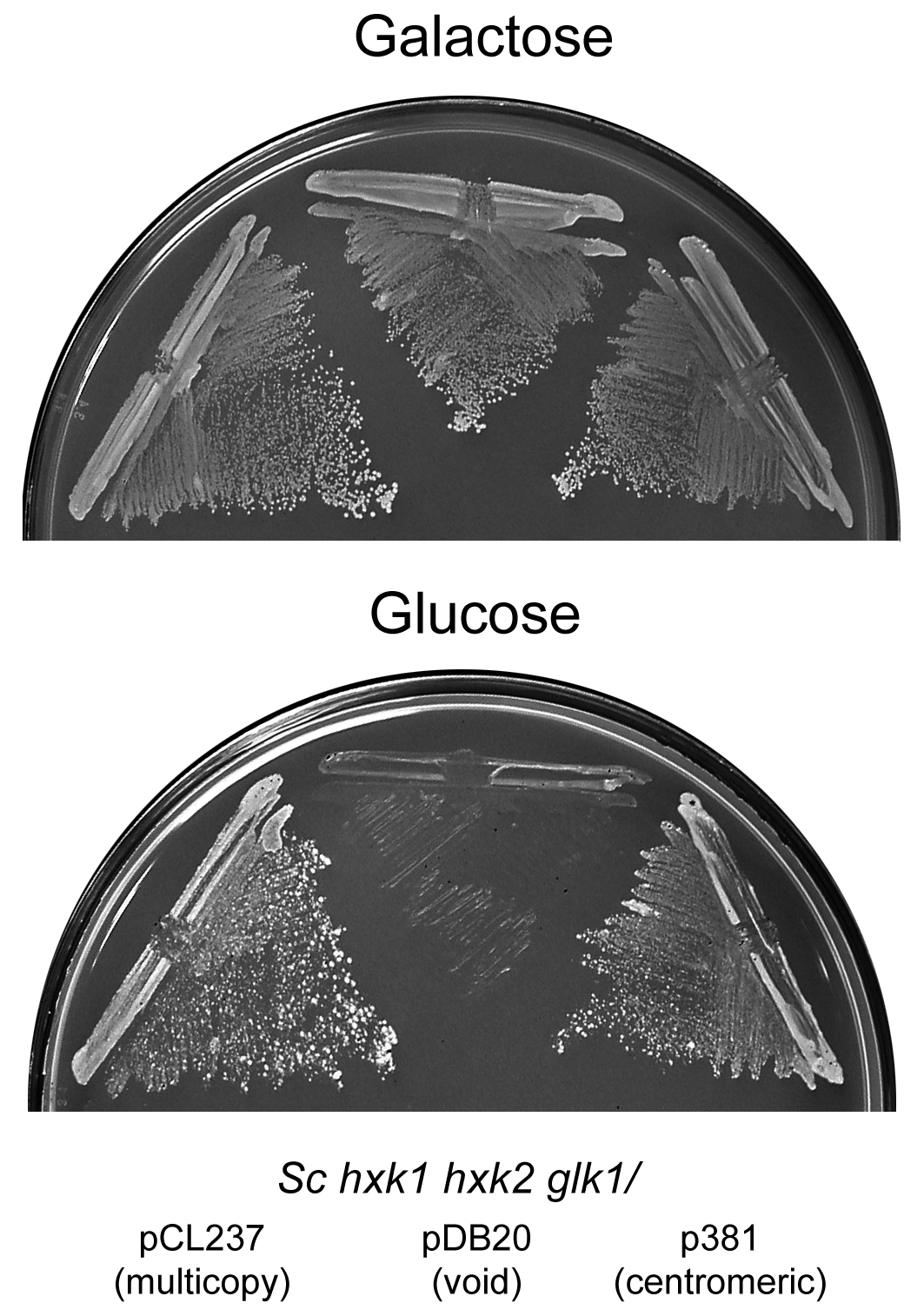

Supplement: S1 Fig — Strain CJM 864 was transformed with plasmids pDB20 (void), pCL150 (YlNAG5-multicopy), and p381 (YlNAG5-centromeric) and streaked in minimal medium with glucose or galactose as carbon sources. (TIF) [file pone.0122135.s001.tif]

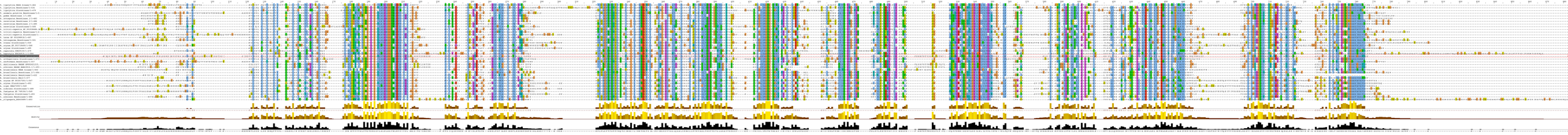

Supplement: S2 Fig — The alignment generated by MEGA was converted to PIR format using the Format Converter v2.3.5 from the HIV Sequence Database (http://www.hiv.lanl.gov/content/sequence/FORMAT_CONVERSION/form.html) and coloured with the ClustalX color code using Jalview (http://www.jalview.org/). (TIF) [file pone.0122135.s002.tif]

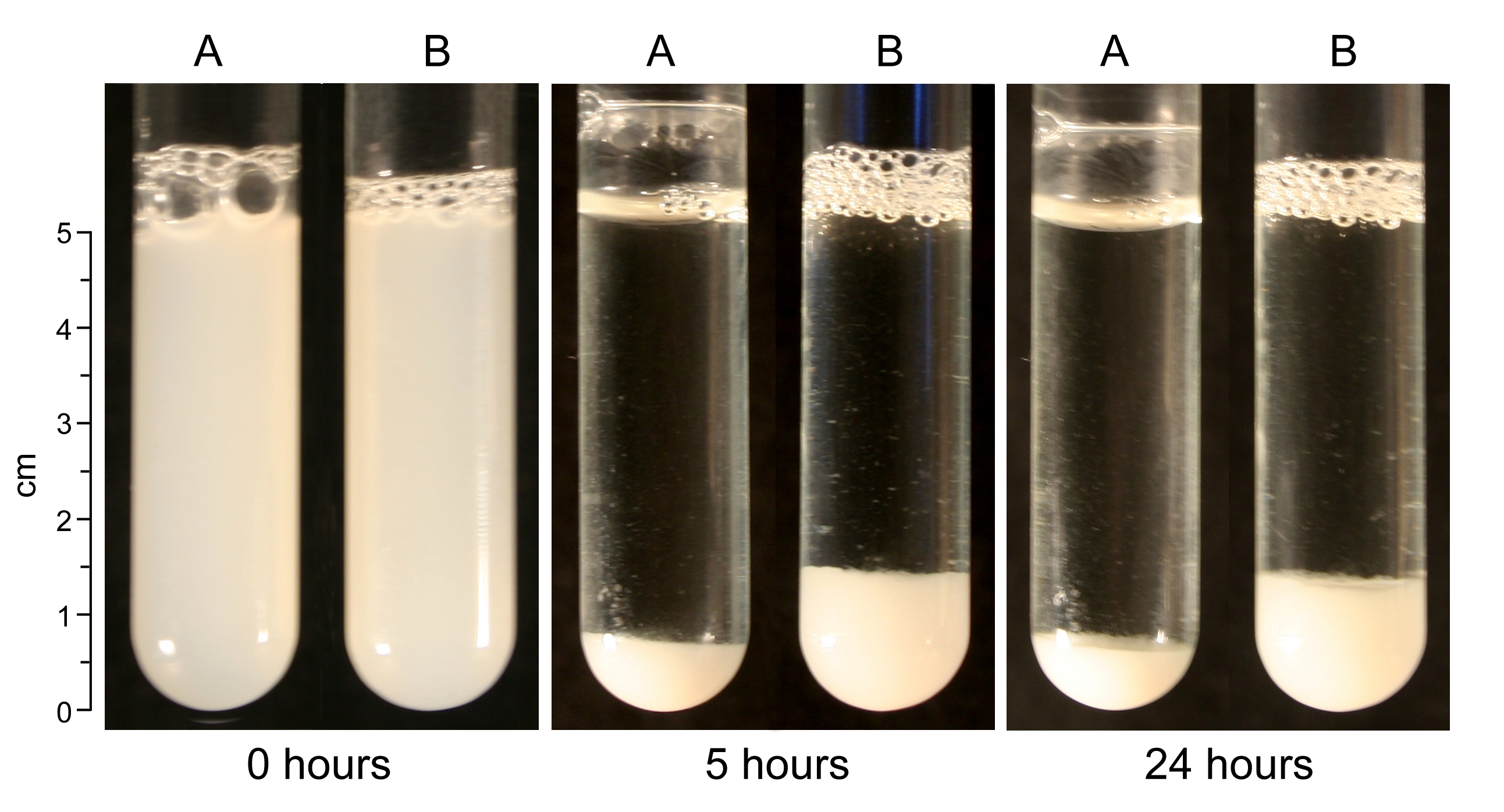

Supplement: S3 Fig — Y. lipolytica strains CJM660 (PO1a/pCL49L, void plasmid) and CJM762 (PO1a/pCL149L-YlNAG5) were grown to an optical density of 10 in minimal medium glucose; 5 ml were transferred to test tubes and photographed after 5h and 24h standing at room temperature. A, CJM660; B, CJM762. (TIF) [file pone.0122135.s003.tif]
