# Supplementary material for: The Gene YALI0E20207g from Yarrowia lipolytica Encodes an N-Acetylglucosamine Kinase Implicated in the Regulated Expression of the Genes from the N-Acetylglucosamine Assimilatory Pathway
Source: PLoS One. 2015 Mar 27;10(3):e0122135. doi: 10.1371/journal.pone.0122135 (PMC4376941; doi:10.1371/journal.pone.0122135)
Supplement: S1 Table — a) Strain CJM 864 was originated as follows: Strains with individual disruptions were crossed, the diploids sporulated and spores were isolated by micromanipulation. Double disruptants of adequate mating type were crossed to select strain CJM 864. This strain was then transformed with the indicated plasmids. b) Plasmid p381 is centromeric and expresses YALI0E20207g under the control of the ScMET25 promoter. (DOC) [file pone.0122135.s004.doc]

**S1 Table. *Saccharomyces cerevisiae* strains used in this work.**

| Strain | Relevant genotype | Origin |
| --- | --- | --- |
| BY4741 | MATa *his3*Δ*1 leu2*Δ*0 met15*Δ*0 ura3*Δ*0* | Euroscarf collection |
| BY4742 | MATα *his3*Δ*1 leu2*Δ*0 lys2*Δ*0 ura3*Δ*0* | Euroscarf collection |
| Y05867 | MATa *his3*Δ*1 leu2*Δ*0 met15*Δ*0 ura3*Δ*0* *hxk1*::*kanMX4* | Euroscarf collection |
| Y04620 | MATa *his3*Δ*1 leu2*Δ*0 met15*Δ*0 ura3*Δ*0* *hxk2*::*kanMX* | Euroscarf collection |
| Y03447 | MATa *his3*Δ*1 leu2*Δ*0 met15*Δ*0 ura3*Δ*0 glk1*::*kanMX* 4 | Euroscarf collection |
| CJM 864 | MATα *hxk1::KanMX4 hxk2::KanMX4 glk1::KanMX4 his3 leu2 ura3* | This work ^a)^ |
| CJM 901 | MATα *hxk1::KanMX4 hxk2::KanMX4 glk1::KanMX4 his3 leu2 ura3* /pCL150 | This work |
| CJM 904 | MATα *hxk1::KanMX4 hxk2::KanMX4 glk1::KanMX4 his3 leu2 ura3 /* pDB20 | This work |
| CJM 910 | MATα *hxk1::KanMX4 hxk2::KanMX4 glk1::KanMX4 his3 leu2 ura3 /* p381 ^b)^ | This work |

a) Strain CJM 864 was originated as follows: Strains with individual disruptions were crossed, the diploids sporulated and spores were isolated by micromanipulation. Double disruptants of adequate mating type were crossed to select strain CJM 864. This strain was then transformed with the indicated plasmids.

b) Plasmid p381 is a centromeric plasmid that expresses *YALI0E20207g* under the control of the *ScMET25* promoter.
