# Supplementary material for: The Gene YALI0E20207g from Yarrowia lipolytica Encodes an N-Acetylglucosamine Kinase Implicated in the Regulated Expression of the Genes from the N-Acetylglucosamine Assimilatory Pathway
Source: PLoS One. 2015 Mar 27;10(3):e0122135. doi: 10.1371/journal.pone.0122135 (PMC4376941; doi:10.1371/journal.pone.0122135)
Supplement: S2 Table — (DOC) [file pone.0122135.s005.doc]

**S2 Table. Primers used for the RT-qPCR assays**

| Target gene | Primer name | Primer sequence |
| --- | --- | --- |
| *NGT1* / *YALI0D09801g* | NGT1-F | 5’-GCGGTCGGATGGTTGATTGC |
|  | NGT1-R | 5’-GTGCTTGTCGGTGACAATGG |
| *NAG5/YALI0E20207g* | YALI0E20207g-F | 5’-AGACCATGGGCAAGGGCTAC |
|  | YALI0E20207g-R | 5’-ACCAGAGACGCCACCGAATC |
| *NAG2* /*YALI0E20163g* | NAG2-F | 5’-TCCAGAACGTCCGTGTCATC |
|  | NAG2-R | 5’-CTTGGCATGGGCATAAGAGC |
| *NAG1* / *YALI0C01419g* | NAG1-F | 5’-TACGAGGCCACCATTGCATC |
|  | NAG1-R | 5’-AAACACCTGTCGGGTTCGAG |
| *GFA1* /*YALI0B21428g* | GFA1-F | 5’-CCTGGAGGATGACGATATTG |
|  | GFA1-R | 5’-ATCTCCTTCTGCATGAAGTG |
| *GNA1* /*YALI0D20152g* | GNA1-F | 5’-GTACTGGCAGGACCGAAACG |
|  | GNA1-R | 5’-CCGATGAGGCCACAGCAATG |
| *AGM1* /*YALI0E29579g* | AGM1-F | 5’-CTCAAGTGCGTTGTTGACTG |
|  | AGM1-R | 5’-CAGCACCACAATCCATGTTC |
| *UAP1* / *YALI0E03146g* | UAP1-F | 5’-TGGAAACGGCGGTCTCTATC |
|  | UAP1-R | 5’-GCACAACCTTGGTGGCAATG |
| *CHS3* / *YALI0C24354g* | CHS3-F | 5’-ACCCTCTCGCCCACACCATG |
|  | CHS3-R | 5’-ATGGGAGTTAGGGTAGTCGG |
